# Protein Thermal Stability Enhancement by Designing Salt Bridges: A Combined Computational and Experimental Study

**DOI:** 10.1371/journal.pone.0112751

**Published:** 2014-11-13

**Authors:** Chi-Wen Lee, Hsiu-Jung Wang, Jenn-Kang Hwang, Ching-Ping Tseng

**Affiliations:** 1 Institute of Bioinformatics and Systems Biology, College of Biological Science and Technology, National Chiao Tung University, Hsinchu, Taiwan, Republic of China; 2 Department of Biological Science and Technology, College of Biological Science and Technology, National Chiao Tung University, Hsinchu, Taiwan, Republic of China; Bioinformatics Institute, Singapore

## Abstract

Protein thermal stability is an important factor considered in medical and industrial applications. Many structural characteristics related to protein thermal stability have been elucidated, and increasing salt bridges is considered as one of the most efficient strategies to increase protein thermal stability. However, the accurate simulation of salt bridges remains difficult. In this study, a novel method for salt-bridge design was proposed based on the statistical analysis of 10,556 surface salt bridges on 6,493 X-ray protein structures. These salt bridges were first categorized based on pairing residues, secondary structure locations, and Cα–Cα distances. Pairing preferences generalized from statistical analysis were used to construct a salt-bridge pair index and utilized in a weighted electrostatic attraction model to find the effective pairings for designing salt bridges. The model was also coupled with B-factor, weighted contact number, relative solvent accessibility, and conservation prescreening to determine the residues appropriate for the thermal adaptive design of salt bridges. According to our method, eight putative salt-bridges were designed on a mesophilic β-glucosidase and 24 variants were constructed to verify the predictions. Six putative salt-bridges leaded to the increase of the enzyme thermal stability. A significant increase in melting temperature of 8.8, 4.8, 3.7, 1.3, 1.2, and 0.7°C of the putative salt-bridges N437K–D49, E96R–D28, E96K–D28, S440K–E70, T231K–D388, and Q277E–D282 was detected, respectively. Reversing the polarity of T231K–D388 to T231D–D388K resulted in a further increase in melting temperatures by 3.6°C, which may be caused by the transformation of an intra-subunit electrostatic interaction into an inter-subunit one depending on the local environment. The combination of the thermostable variants (N437K, E96R, T231D and D388K) generated a melting temperature increase of 15.7°C. Thus, this study demonstrated a novel method for the thermal adaptive design of salt bridges through inference of suitable positions and substitutions.

## Introduction

The need for thermostable proteins has greatly promoted protein engineering in chemical, biotechnological, and food industries [1], [2]. In thermal processes, the use of thermostable proteins exhibits several advantages, including increased productivity, reduced resource depletion, and prevention of microbial contamination. Several studies have been conducted to identify the factors associated with thermal adaptation [3]–[11]. For instance, the thermal stability of proteins is affected by hydrophobicity, packing density [3], [4], number of disulfide bonds [5], strength of electrostatic interactions [6], [7], length of surface loops [4], conformational rigidity [8], [9], amino acid coupling patterns [10], and local structural entropy [11]. These factors not only provide information regarding thermal resistance but pose a great challenge on the development of thermostable proteins.

Electrostatic interaction is one of the most relevant factors in determining protein thermal stability [6], [7], [12], [13]. Furthermore, an increased number of surface charges and salt bridges are observed in thermophilic proteins, in which they contribute to conformational specificity, thermal stability, and oligomerization [14]–[16]. Therefore, increasing electrostatic attraction on protein surfaces was suggested to enhance protein thermal stability [17]–[19]. These stabilizing effects often originate from the increase in protein rigidity and decrease in free energy [20]. The suitable substitutions on protein surfaces can prevent the disruption of conformational changes that occur in a well-packed buried core [6], [21]. Thus, the design of salt bridges on the protein surface is a promising strategy to enhance thermal stability.

Salt bridges in proteins are often formed when two oppositely charged groups interact; in particular, the two charged groups include the cationic ammonium ion of a basic amino acid residue and the anionic carboxylate ion of an acidic amino acid residue spanning a gap of <4 Å. Salt bridges are rarely isolated protein structures; instead, these structures are an integrated part of complex interaction networks that often occur in thermophilic proteins [22], [23]. Therefore, specific geometric conformations are critical in determining the stabilization effect of salt bridges [14], [24]. These geometric conformations depend on the orientation of the side-chains of positively charged residues (e.g. Lysine and Arginine) with respect to the oppositely charged residues (e.g. Aspartate and Glutamate), solvent accessibility, structural context, and local environment, among others [24], [25]. However, rational design of salt bridges to enhance protein thermal stability remains a difficult task because the rules for predicting the positions that result in the effective replacement by charged residues to form salt bridges are still unclear.

B-factor represents the atomic displacement parameters that are obtained from X-ray crystallographic data. This parameter indicates the blurring of atomic electron densities with respect to their equilibrium positions because of thermal motion and positional disorder [26]. Residues with high B-factors often exhibit high flexibility and low thermal stability. Reetz and his group [9] developed an approach coupled with an evolutionary strategy to obtain thermostable proteins by using saturation mutagenesis at the high B-factor residues. Besides, packing density is another important factor that affects protein thermal stability. Our previous studies have shown that the weighted contact number (WCN) can reflect the packing density and is highly correlated with the B-factor [27], [28]. The interaction of two opposite-charge groups to form a salt bridge enhances the rigidity of the connection between two secondary structures where the salt bridge is located, which results to a decrease in local flexibility and an increase in packing density. Thus, protein thermal stability is enhanced [20], [29], [30]. In summary, B-factor and WCN of an amino acid residue are indicators that can be used to design stable salt bridges.

In this study, we developed a method with a scoring system to enhance protein thermal stability based on a salt-bridge design on protein surface. The surface residues, which qualify the criteria of B-factor, WCN and other parameters, were selected. Substitutions that exhibit a high probability of forming a bridge with the surrounding charged residues were computed for each selected position. For example, the polar residues Ser, Thr, Cys, Asn, and Gln are located near negatively charged residues with a high probability of forming salt bridges. Thus, these residues can be mutated to positively charged amino acids, such as Lys or Arg, to form new salt bridges. As a result, electrostatic stabilization is increased. Afterwards, the electrostatic stabilizations from the substitutions (Lys, Arg, Asp, and Glu) of each selected position were compared with the scores that were calculated by using our scoring model.

The production of cellulase required to degrade lignocellulosic substrates constitute a great portion of the total cost of bio-alcohol production [31]. The use of thermostable cellulases in cellulose degradation offers advantages such as reducing the enzyme dosage because of the extension of the enzyme half-life and the capacity to increase the activity at high temperatures [32]. The β-glucosidase A (BglA, EC 3.2.1.21, PDB code: 1BGA [33]) from *Bacillus polymyxa* is a member of family 1 glycosyl hydrolases. The BglA catalyzes the hydrolysis of β-1,4-glucosidic bonds of cellobiose, which is the last step in cellulose degradation. In addition, this enzyme is mesophilic and exhibits a homo-octameric structure [(β/α)_8_] in the native state. Therefore, the BglA can be used as a polymeric model for the salt-bridge design. In this study, our method was applied on the BglA, and six pairs of putative salt-bridges were successfully designed. The experimental results suggested that our method can identify novel salt bridges stabilized by native electrostatic environments to increase protein thermal stability.

## Materials and Methods

### Protein data set for determining geometric preferences of salt bridges

To determine the specific geometric features of salt bridges, a protein structure set was obtained from the Pisces web server with the following criteria: resolution <2.5 Å; *R*<0.25; sequence similarities between any pair protein <25%; and chain length ranging from 60 to 800 residues [34]. The data set comprised of 6,493 X-ray protein structures (Text S1).

### Residue pairs of salt bridges

A salt bridge is formed between oppositely charged residues, such as Arg (R) or Lys (K) and Asp (D) or Glu (E). In this study, His (H) was excluded because this amino acid exhibits an ambiguous protonation state at pH 7.0. Therefore, the following four types of attractive interactions were considered: K–D; K–E; R–D; and R–E. The salt bridge is defined based on the following statements; let atom *A* be 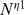 or 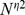 of R or 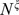 of K, and let atom *B* be 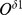 or 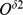 of D or 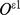 or 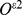 of E. A distance between atoms *A* and *B* less than 4 Å indicates the formation of a salt bridge between *A* and *B*. According to this definition and the considered types of residue pairings, 32,096 salt bridges were isolated from the 6,493 protein structures in the data set.

### Relative solvent accessibility and secondary structure locations of salt bridges

The 32,096 salt bridges were classified into buried or exposed state according to the Relative Solvent Accessibility (RSA) of the salt-bridge residues. RSA is the ratio of the solvent exposed surface area of a residue [35]. To calculate the RSA of a salt-bridge residue, the residue's solvent accessibility (ASA) obtained from DSSP program was divided by a reference value of surface area for the given amino acid (X) in an extended Gly–X–Gly peptide. Salt bridges were considered at the exposed state if the RSA of one residue was >35% and that of the other residue was >25% [36], [37]. After that, the exposed salt-bridges were further categorized according to the located secondary structure and other parameters. The following types of secondary structures are defined in the DSSP program: H, G, I, B, E, T, S, and U [35]. For simplicity, these structures were categorized as helix (H = “H, G, and I”), sheet (S = “B and E”), and coil (C = “T, S, and U”).

### Geometric orientation of salt bridges

**Figure 1:**
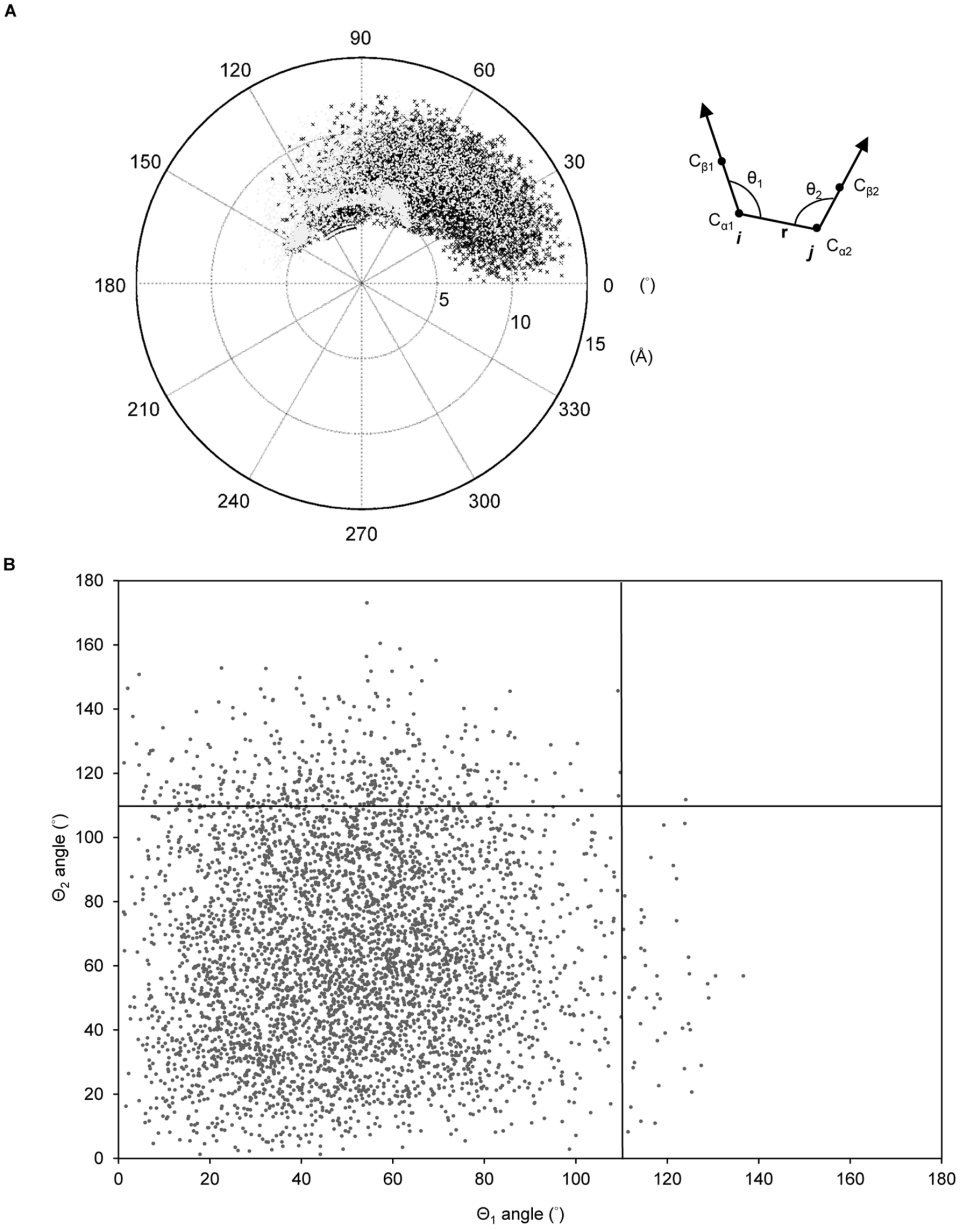
Spatial orientation and Cα–Cα distances of salt bridges on protein surfaces. (A) Statitical analysis of angles (θ_1_, θ_2_) of 10,556 salt bridges on the surfaces of 6,493 X-ray protein structures. Two angles of a salt bridge (*i*–*j*), ∠Cβ_1_Cα_1_Cα_2_ (θ_1_) and ∠Cβ_2_Cα_2_Cα_1_ (θ_2_), were used to describe the charge group interaction based on the relative orientation of the two residues’ Cα–Cβ vectors as indicated. All of the angles are in the range of 0° to 180° (θ_1_ and θ_2_ color in black and gray). The length of radius are corresponding to Cα–Cα distance (Å). (B) The scatter plot shows the angles (θ_1_, θ_2_) of the salt bridges at a backbone distance >7 Å, which are restrained within 0° to 110°.

The Cα and Cβ atomic coordinates of two residues (*i, j*) of a salt bridge were used to determine the angles (θ_1,_ θ_2_). Two angles of a salt bridge (*i*–*j*), ∠Cβ_1_Cα_1_Cα_2_ (θ_1_) and ∠Cβ_2_Cα_2_Cα_1_ (θ_2_), were used to describe the geometric interaction of two oppositely charged groups according to the relative orientation of the two residues’ Cα–Cβ vectors (Figure 1A).

### Salt-bridge pair index of weighted electrostatic attraction model

To find the effective pairings for a salt-bridge design, we developed a weighted electrostatic attraction model 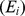, which is a scoring function, to measure the attractive interactions of residue *i* in a protein structure. 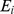 is defined as follows:
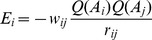
(1)where 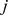 represents the residues within a radius of 15 Å of 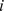; 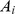 and 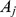 are the amino acids of residues 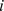 and 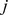, respectively; 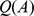 is an electrostatic charge function that depends on the charge of the amino acid: 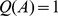 if 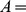
Lys or Arg; 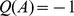 if 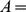
Asp or Glu; and 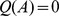 if 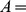
other types of amino acids. 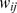 is an empirical parameter generated by the statistical analysis of 10,556 surface salt bridges in the data set. These salt bridges were first classified based on the following parameter set: (1) Cα–Cα distance (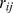) of a salt bridge; (2) oppositely charged amino acid types (i.e., 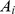 and 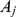); and (3) the secondary structures of residues 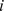 and 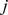 locations. A 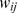 set for each type of salt bridges depends on the frequency of occurrence in the 10,556 surface salt bridges. Therefore, these 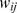 values corresponding to different frequency of specific types of salt bridges could be seen as a salt-bridge pair index to help determine the designable salt bridges with a high probability of formation. In addition, since the repulsive interactions were not taken into consideration, the frequency of repulsive pairs were not analyzed: 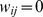, if 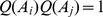.

### Basic rationale of our approach

To design salt bridges on a protein by using our approach, the following procedures were performed. The residues with the following characteristics were isolated: RSA >35%; Z-score of B-factor (z_B-factor) >0; Z-score of rWCN (z_rWCN) >0; and conservation <4. The idea of this pre-filtering was to select the potential residues that can be designed to improve protein thermal stability. Note that only polar residues (Ser, Thr, Cys, Asn, and Gln) and charged residues (Lys, Arg, Asp, and Glu) were considered because polar residues are scarcer in thermophilic proteins compared with their homologous counterparts [38]. The WCN of an atom is the sum of the inverse squares of the distances between the atom and other atoms; rWCN is the reciprocal of WCN [27], [28]. The rWCN value was utilized in the selection of potential residues for designing salt bridges. The rWCN and B-factor of a residue were computed using those values of the Cα atom only. The values were further normalized to their respective Z-scores (z_B-factor and z_rWCN). The normalization was calculated as 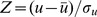, where 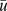 and 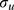 are the mean and standard deviation of 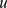. The value of 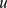 is either the B-factor or the rWCN value. The evolutionary conservation of a residue was also taken into consideration because low conservation residue may indicate less importance in determining the structural conformation and function of proteins [39]. Therefore, low conservation residues can be mutated to design salt bridges without affecting protein folding and activity. The conservation scores obtained from the Consurf data set were used in the selection [39]. Each position fit all of the above criteria was then independently assumed as K, R, D, and E and scored by Eq. (1) to determine the most efficient pairing of each position with one of the surrounding charge residues within a radius of 15 Å. If a multimeric protein complex was obtained, the residues across the interface of another inter-subunit within a radius of 15 Å were also considered. Before the scoring, the surrounding charge residues within 15-Å radius of each position were located. Therefore, the putative pairs of each position can be obtained. If the putative pairs of a position were all at an interval of <5 residues of the primary sequence, they were not scored because the pairings may not be influential for increasing thermal stability. Besides, to ensure that the side chains of the two pairing residues were oriented toward each other, the angles of one pair were restricted to a limited degree. The angular criteria were established from the statistical analysis of the surface salt bridges in the data set and discussed in the results and discussion section. Only the pairs that fit the angular criteria were scored by Eq. (1). The most efficient type of charged amino acid for each position to form the highest-scoring salt bridge was identified to perform site-directed mutagenesis.

### Site-directed mutagenesis and Protein overexpression

A plasmid encoding the BglA (pBglA) was used as a site-directed mutagenesis template. Fifteen pBglA plasmid amplification cycles were performed with the primers containing the mutated nucleotides and KOD DNA polymerase (Takara Inc.). The amplified material was digested with Dpn I and the digested mixtures were used to transform *Escherichia coli* DH5α. Kanamycin-resistant colonies were then isolated on a LB plate (MDBio Inc.) containing 25 µg mL^−1^ kanamycin. The mutated plasmids were extracted and used to transform *E. coli* BL21 Star (DE3) for protein overexpression. Cells were grown in LB broth containing kanamycin (25 µg mL^−1^) at 37°C with shaking to optical density at 600°nm (OD_600_) of 0.4. Isopropyl β-D-1-thiogalactopyranoside (IPTG) of 0.5 mM was added to induce the BglA overexpression overnight at 25°C.

### Protein purification

Cell destruction was performed using an Avestin Emulsiflex Homogenizer EF-C3 (Utek Process) under 20,000 psi pressure for five cycles. Insoluble cell debris was removed by centrifugation at 16,000×*g* for 15°min at 4°C. The supernatant was passed through a HisTrap column (GE Healthcare Inc.). After the column was washed with a phosphate binding buffer (10 mL, 20°mM) containing imidazole (20 mM), elution was performed using 20°mM phosphate buffer containing imidazole (100 mM). The elution buffer was replaced with 20°mM PBS buffer by using a HiTrap desalting column (GE Healthcare Inc.). His-tag was removed by incubating the purified protein with thrombin (GE Healthcare Inc.) at 20°C for 10°h. The resulting sample was reloaded on the HisTrap column. The eluted protein was collected and concentrated using a 10,000 MWCO centrifugal filter (Millipore Inc.). The concentrated protein was purified by gel filtration (HiLoad 26/60 Superdex 200 prep-grade, GE Healthcare Inc.) using citrate phosphate buffer (50 mM, pH 7) to elute 400 kDa BglA.

### Thermal denaturation and kinetic studies of BglA and mutants

The thermal stability and kinetics of the purified BglA and mutants were measured. The melting temperature (*T*_m_) was determined by differential scanning calorimeter (VP-DSC, GE Healthcare Inc.) with protein samples (2.0 mg/mL) in a solution of 50°mM citrate phosphate buffer at pH 7.0. The scanning temperature ranged from 20°C to 70°C at a heating rate of 1°C/min. The resulting data were fitted to non-2-state model. 4-Nitrophenyl β-D-glucopyranoside (pNPG; Sigma) was used as an artificial substrate for detecting β-glucosidase activity. The kinetic assays were conducted at 37°C for 5.5 minutes in 50°mM citrate phosphate buffer, pH 7.0, using 1 µg.800 µl^−1^ enzyme and 0.02−3 mM pNPG, modified based on the previously described method [40]. The results were fit to the Michaelis-Menten equation for determination of the *K*_m_ and *k*_cat_ by the software of GraphPad Prism 5.

## Results and Discussion

### Geometry preferences of salt bridges

In total, the data set consisted of 32,096 salt bridges from 6,493 X-ray protein structures. To analyze the specific geometry of salt bridges on the protein surface, we further classified these salt bridges as a buried or exposed state based on the RSA value (Table 1). The analysis revealed that the K–E pairs favored the formation of exposed salt bridges, whereas R–D pairs were more common in buried salt bridges. The result may suggest that stability of K–E pairings were higher on protein surfaces than in buried regions. Hence, the analyses were consistent with those in previous reports, in which K and E are more common on the surface of thermophilic proteins than on mesophilic homologues [38]. The 10,556 salt bridges at the exposed state were regarded as surface salt bridges and used for the following statistical analysis (Table 1).

**Table 1:** Relative solvent accessibility of salt bridges in 6,493 X-ray protein structures.

Location	Salt-bridge pair				Total
(RSA)	K–D	K–E	R–D	R–E	
All	8040	9479	7240	7337	32096
(≥0%)^a^	25.00%	29.50%	22.60%	22.90%	
Buried	657	525	750	730	2662
(<9%)^a^	24.70%	19.70%	28.20%	27.40%	
Exposed	2947	3764	1742	2103	10556
(>35%, >25%)^b^	27.90%	35.70%	16.50%	19.90%	

Abbreviation: K, Lysine; R, Arginine; D, Asparagine; E, Glutamine; and RSA: Relative solvent accessibility.

a RSA of both residues of a salt bridge are indicated.

b RSA of one residue of a salt bridge is >25% and the other is >35%.

Figure 1A shows the Cα–Cα distance and angular distributions of the 10,556 surface salt bridges. The angles of the surface salt bridges ranged from 0° to 180°, which could be used as an angle constraint. The longest Cα–Cα distance was observed in the R–E pair in PDB 1HBN (R535–E39 coil–helix at 14.235 Å, θ_1_∶27.16°, and θ_2_∶9.45°), whereas the shortest was observed in the K–D pair in PDB 1D2T (K90–D91 coil–coil at 3.719 Å, θ_1_∶97.08°, and θ_2_∶119.25°). The results suggested that the range of possible interaction in a salt-bridge extended to 15 Å of Cα–Cα distance approximately. Hence, we set a 15-Å radius of a position as the considered area in the search for the surrounding charge residues. Figure 1B shows the angles (θ_1_, θ_2_) of the salt bridges with Cα–Cα distance >7 Å. Based on statistical analysis, the two angles (θ_1_, θ_2_) of 89.7% salt bridges were both restrained within 0° to 110° when the Cα–Cα distance (*r*) was >7 Å. For the remaining 10.2% salt bridges, when one of the angles was >110°, then the second angle should be <110°, with only one exception. Therefore, we set the angular restraint of <110° for at least one angle (θ_1_ or θ_2_) if the Cα–Cα distance is >7 Å to ensure the close proximity of the ionic groups of the salt bridges to each other.

**Figure 2:**
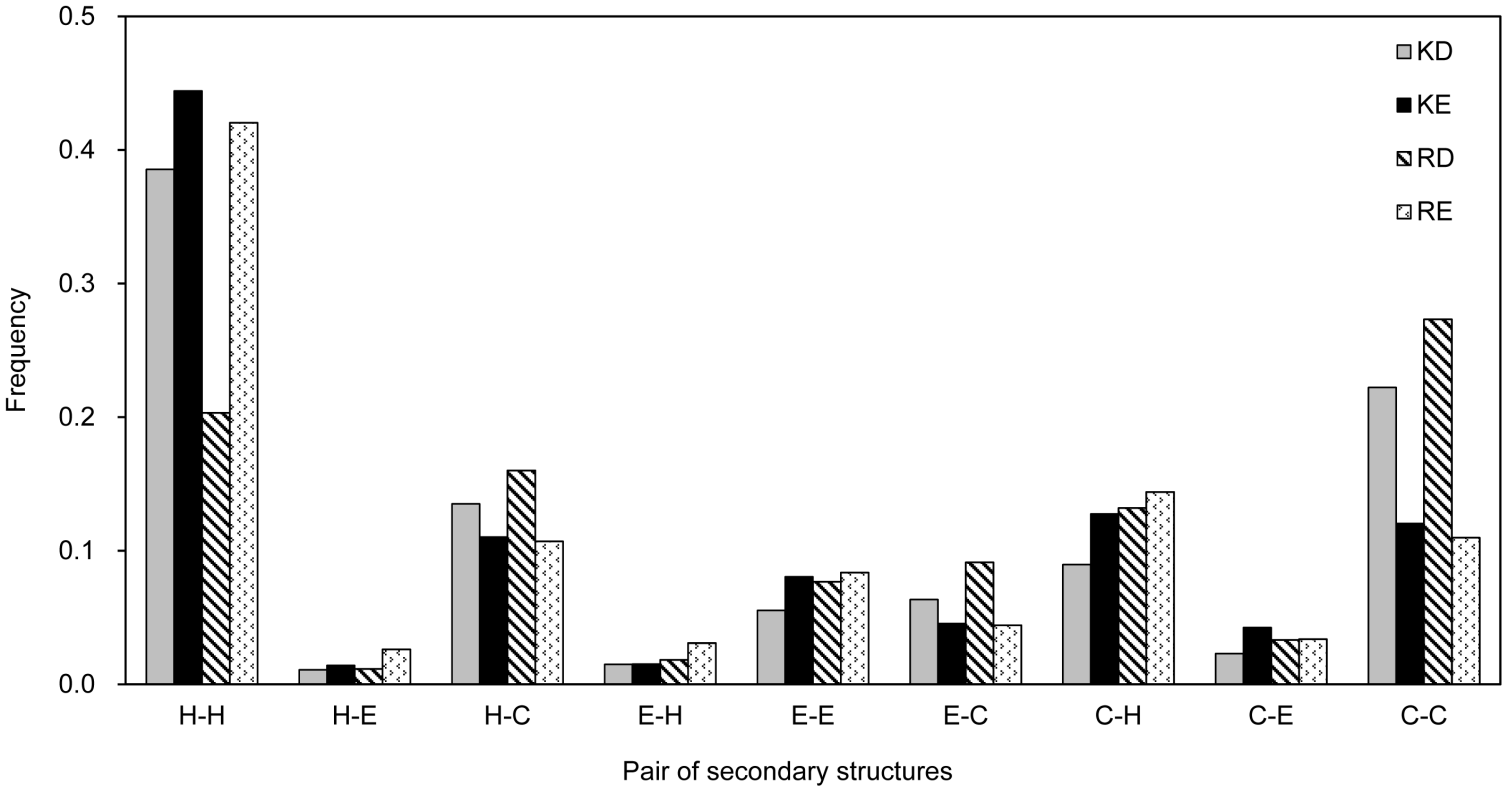
Frequency distributions of surface salt bridges at different pairs of secondary structures. Four types of salt-bridge pairings, Arg/Asp (R/D), Arg/Glu (R/E), Lys/Asp (K/D), and Lys/Glu (K/E), were considered in the statistical analysis.

**Figure 3:**
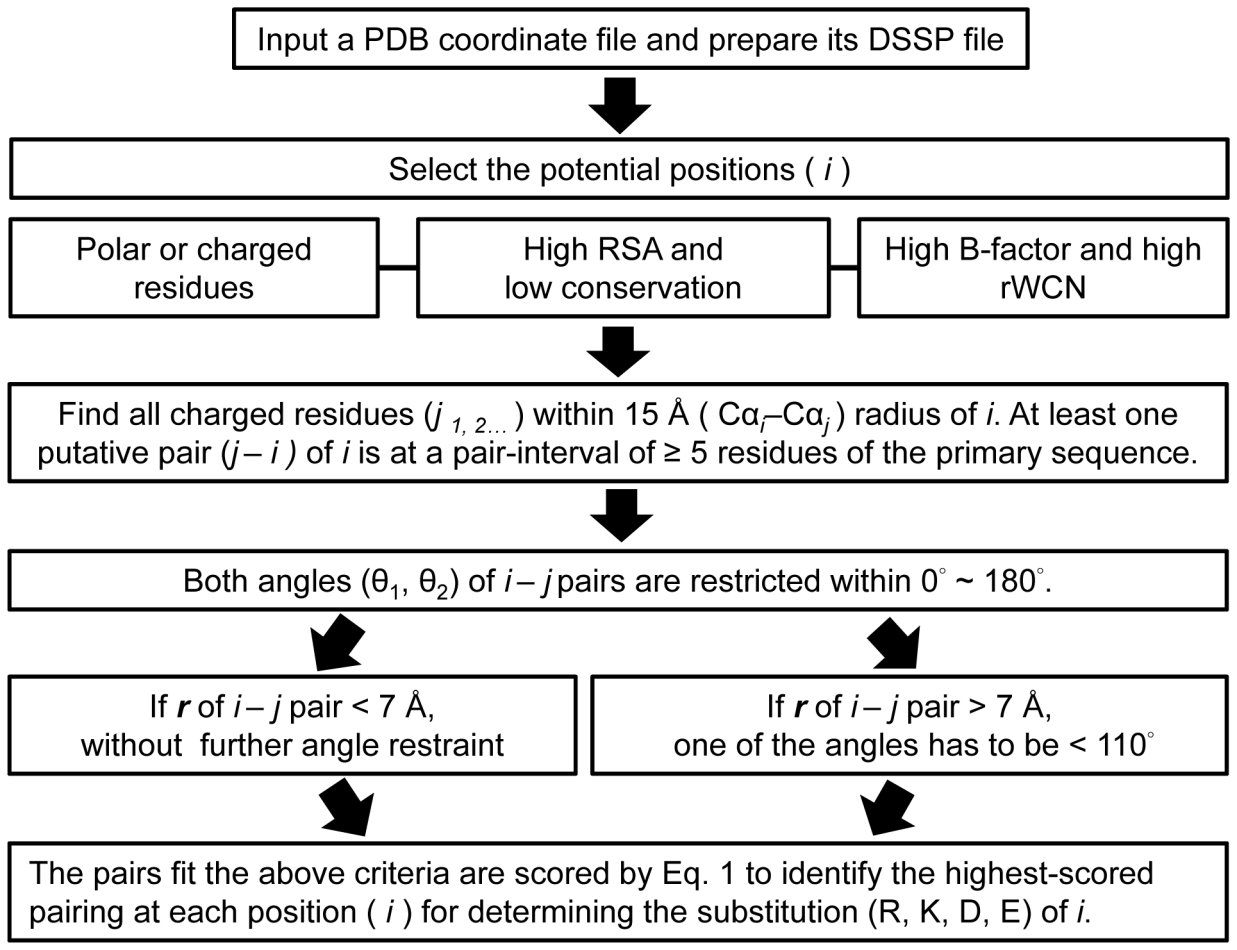
Flowchart of salt-bridge design in this study. Identification of potential positions and mutation types for a given protein structure are demonstrated.

We further classified the surface salt bridges based on the secondary structure locations. Figure 2 shows the frequency distributions of K–D, K–E, R–D, and R–E salt-bridges corresponding to different secondary structure pairs. The highest frequency occurred at the helix-helix structure for all pairings of the salt bridges except the R–D pairings. Thus, such salt bridge formation at the helix-helix structure may efficiently stabilize protein structures. Based on previous studies, the formation of R–E salt-bridge at the helix−helix structure may account for much of the stabilization of certain thermophilic proteins [41]. To determine the exact value standing for the preference of salt bridge formation of a particular type of ion pair, we constructed a matrix of empirical weights (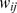) based on the frequency of each type of salt bridges. To calculate the frequency of each type of salt bridges, the 10,556 surface salt-bridge pairs were first classified based on pairing residues, Cα–Cα distances, and secondary structure locations. Table S1 shows the empirical weight of each salt bridge type depending on the frequency. The weight matrix was then used in the weighted electrostatic attraction model to score each pairings. Finally, the angular constraints and the weighted electrostatic attraction model could be used in the last steps of identifying the pairings and the substitutions after the selection of potential positions for salt-bridge design (Figure 3).

### Potential pairings in intra- and inter-subunits

The BglA obtained from *B. polymyxa* was used as a polymeric model for the design of inter- and intra-salt bridges to enhance thermal stability. Polar and charge residues were considered as the pre-filtering criterion in the isolation of potential positions for different reasons. Polar residues are reported to be scarcer in thermophilic proteins, and charged residues can be selected for charge optimization; for example, to avoid electrostatic repulsion [38], [42]. Proteins from thermophiles and hyper-thermophiles exhibit more frequently networked salt bridges than proteins from the mesophilic counterparts [43]. Hence, increasing the thermal stability of proteins by optimization of charge–charge interactions is a good strategy for an evolutionary solution. Besides, the criteria of RSA >35%; z_B-factor >0; z_rWCN >0; and conservation <4 were also used as pre-filters for isolation of residues on the surface of a protein, conformational fluctuation above the average, packing density below the average, and low conservation, respectively [27], [28], [36], [39]. The characteristics used in the pre-filtering of all the 447 BglA residues are shown in Table S2. Thirty four positions that fit the pre-filtering criteria in the BglA were isolated (Pre-filters: STCNQ/KRDE: 185, RSA >35%: 67, z_B-factor >0∶50, z_rWCN >0∶49, and conservation <4∶34) as shown in Table S3. To determine the potential pairings for each 34 positions, we located the charge residues within a radius of 15 Å of the position. The putative pairs of the 34 positions were separately identified as in Table S4. Only 11 positions that could be paired with the surrounded residues were found according to the angular constrains of θ_1_ and θ_2_ generated from the statistical analysis of the surface salt-bridges (Figure 1) and at least one pair for each position that met the sequence separation criteria (≥5 residues). The putative pairs for each 11 positions were separately identified and scored using Eq. (1). To demonstrate how to identify the charge amino acids for substitutions at the 11 positions, Table S5 shows the theoretical scores of the putative pairings between each of the 11 positions and their surrounded charge residues. The predicted pair equivalent to the highest-score pair of each position was the pair with the highest preference for salt-bridge formation. The suggested amino acids for mutations were the one that fit the predicted pair at each position. For example, the suggested mutations would be E96K because E96K–D28 is the predicted pair of E96 position. However, if a position’s highest-scoring pair exhibited a short sequence separation (i.e. <5 residues), the corresponding mutation was not adopted. Moreover, an alternative charged substitution was performed when that substitution for alternative pair formation (i.e., sequence separation ≥5 residues) was scored higher (i.e., higher preference for forming the salt bridge) than the same substitution for inefficient pair formation (i.e., sequence separation <5 residues). The highest-scoring pair was inferred when two or more of such alternative pairs were found. One example in Table S5 was the S440E–R436 pair. The pair exhibits a pair interval <5 residues of the primary sequence and was the highest-scoring pair for the R436 position. Moreover, the S440R–E70 pair’s score was higher than that of the S440R–R436 pair. Therefore, the suggested S440 mutation was S440R for the S440R–E70 pair formation.

**Figure 4:**
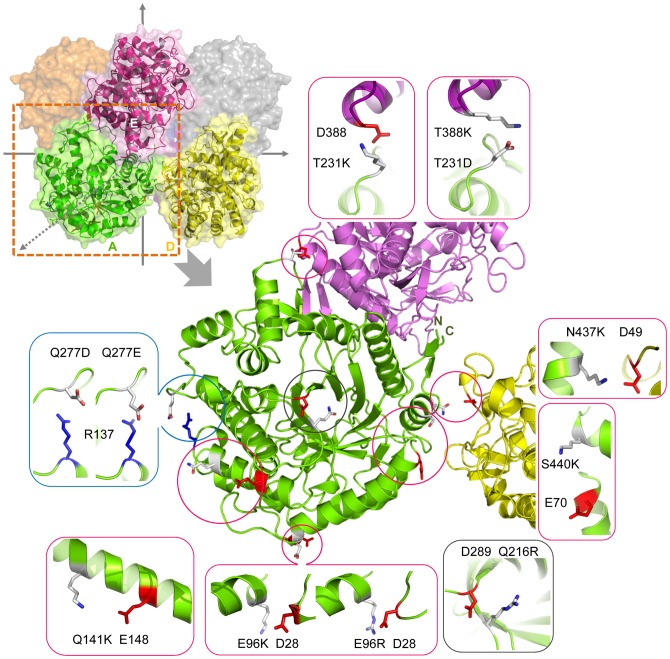
Locations of eight predicted pairs and one control pair in the octameric structure (PDB: 1BGA). A, D, E subunits are represented in green, yellow, and purple, respectively. The N437–D49 pair is between subunits A and D; the T231–D388 pair is between subunits A and E; whereas E96–D28, Q141–E148, Q277–R137, S440–E70 and control pair Q216-D289 are intra-subunit pairs. The predictive pairs as well as T231D–D388K and Q216R–D289 pairs were simulated by Pymol program. Each pair is magnified in an independent window showing secondary structure elements and the paired residues (red for negatively charged residues and blue for positively charged residues). The neighboring charge residue of each position (E96, Q141, T231, Q277, N437, S440, and Q216) suggested the oppositely charged substitutions for forming salt bridges.

Six of the eleven positions in Table S5 (i.e., N47, N88, Q95, T273, E360, and N437) exhibited their highest-scoring pairs to be the pairs with a short sequence separation (<5 residues). Moreover, no alternative substitutions were found for these positions except for the N437 position. Besides, two positions, E96 and Q277, each was found with two suggested mutations for two predicted pairs that showed equal scores (Table S5). Hence, eight mutations for the eight predicted pairs consisted of two inter-subunit pairs (i.e., T231K–D388 and N437K–D49) and six intra-subunit pairs (i.e., E96K–D28, E96R–D28, Q141K–E148, Q277D–R137, Q277E–R137, and S440K–E70) were constructed to perform the experimental thermal stability test. In addition, to validate the pre-filtering criteria set for the salt-bridge design, the Q216 was used as a negative control position because the RSA, z_B-factor and z_rWCN values of Q216 are not qualified but close to the criteria. The z_B-factor and z_rWCN of the Q216 are close but less than 0 and the RSA is less than 35% as shown in Table 2. The D289 was the qualified charge residues to bridge with the Q216R (Cα–Cα distance: 5.52 Å; scores of D289–Q216K/R/D/E: 0.04/0.07/0/0; θ_1_ and θ_2_ of Q216R–D289 are 60.36°°and 11.61°) as shown in Table S5. Therefore, the *T*_m_ resulted from the Q216R–D289 pair was expected not to be higher than that of the wild type enzyme. Figure 4 shows the locations of the eight predicted pairs and Q216R–D289 pair in the BglA structure. Two residues (T231, N437) at the A/D and A/E interfaces and four residues (E96, Q141, Q277, and S440) of the intra-subunit pairs, as well as Q216, were subjected to site-directed mutagenesis. The eight suggested mutations of the six positions (E96, Q141, T231, Q277, N437, and S440) were E96K, E96R, Q141K, T231K, Q277D, Q277E, N437K, and S440K. The structural characteristics of the seven positions (E96, Q141, T231, Q277, N437, S440, and Q216) are summarized in Table 2. All the six positions (E96, Q141, T231, Q277, N437, and S440) qualify the following characteristics of the pre-filtering criteria: polar or charged residue, RSA >35%, z_B-factor >0, z_rWCN >0, and conservation <4. Figure S1 shows the z_rWCN and the z_B-factor profiles of 1BGA: chain A, which demonstrate that the z_rWCN and z_B-factor of the six positions are higher than the average. On the contrary, the Q216, used as a negative control position, exhibits the z_B-factor and z_rWCN slightly lower than the average. The profiles also demonstrate that Q216 is a relatively stable and buried position around the local sequence region.

**Table 2:** Characteristics of positions in BglA for mutations.

Position	SS	RSA	z_B-factor	z_rWCN	Conservation	Location (subunit)
E96	H	0.46	2.46	0.81	3	Intra (A)
Q141	H	0.70	0.16	0.11	3	Intra (A)
Q277	C	0.52	1.12	1.96	2	Intra (A)
S440	H	0.54	0.43	0.66	2	Intra (A)
T231	C	0.96	0.91	3.28	1	Inter (A/E)
N437	H	0.54	0.29	0.53	1	Inter (A/D)
Q216	E	0.16	−0.14	−0.19	4	Intra (A)

Secondary structure (SS: H, helix; E, beta-sheet; C, coil), relative solvent accessibility (RSA), Z-scores of B-factor (z_B-factor) and reciprocal of weighted contact number (z_rWCN), conservation score, and inter- or intra- subunit location are indicated.

### Thermal stability and kinetics of BglA mutants

The Δ*T*_m_ of the enzymes was obtained from DSC experiments, in which the Δ*T*_m_ values and the melting curves of the wild type and the mutants are compared in Table 3 and Figure S2, respectively. The irreversible denaturation of the enzymes could cause the uneven baselines of the melting curves (Figure S2). Therefore, the melting temperatures described below were apparent melting temperatures. Six of the eight suggested mutants showed greater stability than that of the wild type based on the Δ*T*_m_ value (i.e., E96K, E96R, T231K, Q277E, N437K, and S440K) while the two mutants (Q141K and Q277D) showed similar *T*m as that of the wild type. The Δ*T*_m_ caused by the six suggested mutations were 3.7, 4.8, 1.2, 0.7, 8.8, and 1.3°C (E96K, E96R, T231K, Q277E, N437K, and S440K, respectively). Each predicted position that successfully increased the *T*m of the BglA through the suggested mutations was individually mutated to all the four charged amino acids, K, R, D, and E, via site-directed mutagenesis. These additional mutations were conducted to further confirm the predicted accuracy and investigate the relation between the increase of melting temperature (Δ*T*_m_) and the predicted score of each mutant. The Δ*T*_m_ values resulted from the suggested charged mutations that leaded to increased *T*_m_ and the same type of charge mutation at each of the E96, T231, Q277, N437, and S440 positions were higher than that of the opposite type of charge mutations except the Q277D and Q277R mutations (Table 3). The latter two mutations displayed a similar *T*m. These findings suggested that the prediction of the pairings according to the scores calculated by Eq. (1) of the K, R, D, and E substitutions at each five positions were in agreement with the Δ*T*_m_ results. Besides, the *k*_cat_/*K*_m_ values of the thermostable mutants were also measured as in Table 3. Moreover, the mechanisms of thermal adaptation of each position (E96, Q216, T231, N437, Q277, and S440) were varied and discussed in the following sections.

**Table 3:** Δ*T*_m_ and kinetic parameters of wild-type BglA and mutant enzymes.

No.	Mutants	Δ*T*_m_ (°C)	*k*_cat_ (sec^−1^)	*K*_m_ (mM)	*k*_cat_/*K*_m_ (sec^−1^ mM^−1^)
0	Wild-type	0.0^a^	44.73±0.81	1.21±0.03	36.89±0.38
1	E96K^b^	3.7	28.42±0.15	0.19±0.01	149.00±4.97
	E96R^b^	4.8	21.19±0.50	0.47±0.02	44.81±1.08
	E96D	−1	n.d.	n.d.	n.d.
2	Q141K^b^	−0.3	n.d.	n.d.	n.d.
3	Q216K	–0.5	30.62±0.54	0.47±0.04	64.90±4.90
	Q216R	–3.5	26.02±0.12	0.46±0.00	56.87±0.19
	Q216D	0.1	n.d.	n.d.	n.d.
	Q216E	−2.2	n.d.	n.d.	n.d.
4	Q277K	0.4	n.d.	n.d.	n.d.
	Q277R	0.0	n.d.	n.d.	n.d.
	Q277D^b^	−0.1	1.04±0.02	18.06±0.29	17.41±0.12
	Q277E^b^	0.7	0.85±0.31	15.68±3.50	19.00±2.72
5	T231K^b^	1.2	36.32±1.36	0.61±0.07	59.31±4.96
	T231R	−0.3	n.d.	n.d.	n.d.
	T231D	−3.8	n.d.	n.d.	n.d.
	T231E	−3.2	n.d.	n.d.	n.d.
	T231D/D388K	4.8	32.76±0.76	0.19±0.01	174.90±9.48
6	N437K^b^	8.8	34.85±0.18	0.50±0.01	69.74±1.45
	N437R	5.3	10.27±0.51	0.40±0.06	25.57±2.39
	N437D	−0.4	n.d.	n.d.	n.d.
	N437E	−0.4	n.d.	n.d.	n.d.
7	S440K^b^	1.3	1.63±0.03	24.39±0.30	14.99±0.14
	S440R	2.4	3.96±0.32	31.78±1.90	8.04±0.17
	S440D	−0.7	n.d.	n.d.	n.d.
	S440E	0.5	n.d.	n.d.	n.d.
8	E96R/T231D/D388K/N437K	15.7	10.27±0.51	0.40±0.06	25.57±2.39

a *T*_m_ of wild-type protein was detected to be 38.5°C.

b Suggested mutation.

n.d.: Not determined.

### Case 1: E96, reversing the negatively charged residue

The E96 residue exhibits high B-factor and rWCN values (z_B-factor: 2.46, z_rWCN: 0.81), which indicate a large fluctuation and a low packing density, respectively (Table 2). Based on Table S5, E96 in a helix revealed the highest probability of forming an intra-molecular salt bridge with a neighboring D28 residue in a loop structure by reversing from negatively to positively charged K or R residues. The D28 residue is also in a flexible region as indicated by the z_B-factor. Therefore, the bridging of K96 or R96 with D28 could stabilize two flexible regions to increase protein rigidity. In previous studies, the E96K mutation was also detected by random mutation [44]. The mutant was crystallized to demonstrate the formation of a link between K96 and D28 [45]. This crystalline structure also indicates a twist conformation caused by E96K mutation, where the K96 side chain turns to the D28 side chain. We also found that Δ*T*_m_ caused by E96R mutation was even higher than that of E96K mutation. The E96R mutant showed a Δ*T*_m_ of 4.8°C compared with 3.1°C of E96K (Table 3). On the contrary, when a negatively charged Aspartate was introduced at E96, the *T*_m_ of the BglA dropped 1°C. Thus, thermal adaptation occurred more effectively in this environment than in the original environment in the event of reversal of a negatively charged residue (E96) to its positively charged residues (K96 or R96). Previous research has also mentioned that charge reversal of a residue could convert a destabilizing effect to a stabilizing effect because charge reversal alters the core of the interactions; as a result, such destabilized interactions are stabilized [42]. A four-fold increase of *k*_cat_/*K*_m_ value was found in E96K mutant, whereas that of E96R mutant remained almost unchanged.

### Case 2: Q216, with low B-factor and WCN at a slightly buried state

The Q216 residue at a slightly buried state exhibits lower RSA, B-factor, and rWCN values (RSA: 0.16, z_B-factor: −0.14, and z_rWCN: −0.19) than that prescribed in the pre-filtering criteria (Table 2); the surrounded D289 could be paired with the Q216R according to the scores calculated by Eq. (1). Thus, Q216R–D289 was used as a negative control pair. The Δ*T*_m_ of Q216 mutants were lower or similar than that of the wild type enzyme, which was consistent with our predictions (Table 3). Thermal stability of the Q216D and Q216K variants showed 0.1 and −0.5°C of Δ*T*_m_ while a large decrease in *T*_m_ of Q216E and Q216R (−2.2 and −3.5°C) mutants were found, which can be explained by the repulsion that arised from the larger side chains of E and R than that of D and K. The large decrease in *T*_m_ caused by the Q216E and Q216R mutations could be expected from the slightly buried and crowded position indicated by the low RSA and z_rWCN of Q216. Therefore, the Δ*T*_m_ of the Q216R mutant was in agreement with the pre-filtering criteria set for the isolation of designable positions. Bleicher et°al. have proposed that the existence of salt bridges in the hydrophobic core of a mesophilic laminarinase (versus its absence in a hyperthermophilic laminarinase) may be responsible for the differences in the thermal stability between the two enzymes [46]. In the mesophilic enzyme, the existence of charge-charge interactions permeating the hydrophobic core of the enzyme actually destabilizes the structure by facilitating water penetration into hydrophobic cavity. These findings could support the Δ*T*_m_ result caused by the putative pairing of Q216R–D289 in this study.

### Case 3: T231, polarity reversal of a putative electrostatic pair

The T231 residue locates at a coiled structure between two subunits of A and E subunits shows a large rWCN value (z_rWCN: 3.28) in Table 2. When an exposed polar T231 residue was mutated into a positively charged residue (T231K), *T*_m_ was slightly increased (Δ*T*_m_: 1.2°C), whereas the T231R mutant showed a similar thermal stability as that of the wild type enzyme (Table 3). On the contrary, the *T*_m_ of T231D and T231E mutants was 3.8 and 3.2°C lower than that of the wild type. Originally, K231 or R231 was expected to form a salt bridge with D388 across the interface (Table S5). We suspected that the small increase in *T*_m_ caused by T231K mutation was triggered by an intra-subunit electrostatic interaction instead of an inter-subunit electrostatic interaction. Further considering the local environment, we found that the positively charged residue K231 could stabilize the negatively charged environment and interact with one of the three negatively charged residues (E233, E234, and D235) from the neighboring helix of the same subunit at Cα–Cα distances of 6.6, 8.5 and 7.0 Å, respectively. The three negatively charged residues are also closely located to 231 position in the primary sequence. Hence, these negatively charged residues could interfere the expected inter-subunit attraction (T231K–D388) by drawing the positive charge of K231 side chain away from the negative charge of D388 side chain in the opposite direction. Therefore, we hypothesized that the reversal of the charges of the two residues, that is, K231 and D388 into D231 and K388, is more likely to promote the salt bridge formation at the interface due to the interferences are dismissed. Thus, the double mutant (T231D–D388K) was constructed to investigate the hypothesis, which resulted in a large increase in *T*_m_ of 4.8°C. This change was significantly greater than that of T231K mutation (Table 3). The results suggested that the negatively charged residues closely located at the helix next to residue D231, may hinder the flexibility of D231 and drive the side chain of D231 to rotate to cross the interface, which formed a bridge with the newly reverse-charged variant K388 at another subunit. Therefore, the *T*_m_ of T231K and T231D–D388K mutants may also potentially demonstrate that an inter-subunit electrostatic interaction could increase protein thermal stability more efficiently than an intra-subunit interaction of polymeric proteins. A five-fold increase in the *k*_cat_/*K*_m_ value caused by the T231D–D388K mutation was observed, whereas the *k*_cat_/*K*_m_ value of T231K remained almost unchanged (Table 3). The results suggested that the inter-subunit interaction may have influenced the enzyme kinetics of the BglA.

### Case 4: N437, elongating the side chain across the interface

The N437 residue locates in a helix structure was predicted to form an inter-subunit salt bridge with the D49 residue in a coil structure between the A and D subunits by the N437K substitution (Table S5). The N437K and N437R mutations resulted in an increase in *T*_m_ of 8.8 and 5.3°C, respectively (Table 3). The N437K mutation was also found as a thermostable mutation by random mutation in a previous study [44]. Based on the BglA structure [33], there is no direct interaction between the N437 residue of subunit A and D49 of subunit D. In addition, the distance between the amide group of the N437 side chain and the carboxyl group of the D49 side chain is 4.5 Å which is longer than 4 Å required for the salt-bridge formation. Therefore, the substitution of R437 or K437 for N437 may shorten the distance by elongating the length of the residue side chain. Therefore, the putative salt bridge between K437 or R437 and D49 may form across the interface by extending the range of the side chain interaction. By contrast, the *T*_m_ values of N437D and N437E mutants were lower than that of the wild type. During the BglA purification process, the protein gel-filtration data revealed that the N437D and N437E mutants both eluted differently sized tetramers from the native octamer (data not shown). Hence, this position was suggested to function crucially in protein oligomerization.

### Case 5: Q277 and S440, introducing intra-subunit pairings at long distances

The Q277 in a coil structure was predicted to form an intra-subunit salt bridge with the R137 in a helix structure through the Q277D or Q277E mutation (Table S5). The predicted pairs (Q277D–R137 and Q277E–R137) exhibit a 10.96 Å Cα–Cα distance. The Q277E mutant showed a higher *T*m (Δ*T*_m_: 0.7°C) than that of the Q277D mutant (Δ*T*_m_: −0.1°C) (Table 3). Hence, the results suggested that instead of aspartate, a longer glutamate side-chain was more suitable to reach the R137 side chain.

The S440 and E70 residues at a long interval (Cα–Cα: 10.31 Å) were also predicted to interact with each other through the S440K mutation (Table S5). Both the S440K and S440R mutations resulted in increased Δ*T*_m_ values (i.e., 1.3 and 2.4°C, respectively) (Table 3). However, both mutations caused a 0.5-fold drop of *k*cat/*K*m. A decrease of *k*cat/*K*m caused by the Q277E and Q277D mutations was also observed.

### Case 6: Combining the thermostable mutations

The combination of the E96R, T231D–D388K, and N437K mutations with each showed an increased *k*cat/*K*m value compared with that of the wild type provided a maximum Δ*T*_m_ of approximately 15.7°C (Table 3). The large increase in *T*_m_ observed in the combined mutant emphasized the importance of increasing the number of salt bridges in enhancing protein thermal stability [38]. The *k*_cat_/*K*_m_ value was only slightly less than that of the wild type at 37°C.

Previous simulation studies of mesophilic and hyper-thermophilic proteins have shown that protein unfolding may be initiated at sites prone to large thermal fluctuations; an example of such sites include the loop region in rubredoxin [47], [48], whose flexibility allows water to access and unfold the protein structure. In the present study, the novel salt bridges were constructed within the flexible region to minimize fluctuations; hence, the development of resistance to high temperatures was promoted [49]. Previous reports have also indicated the relevance of high rigidity as a factor that influences the integrity of the native folded structure by increasing the packing density on a protein surface [27]–[29], which is consistent with the strategy of constructing salt bridges at positions of low contact number and high solvent accessibility in this study.

The two mutation pairs, T231D–D388K and N437K–D49, in the A/E and A/D inter-subunit could increase multimeric stabilization. The Δ*T*m of T231D–D388K mutant was 4.8°C, and that of N437K–D49 was 8.8°C higher than that of the wild type. According to the previous studies, inter-subunit interactions are known to preserve oligomer structures and increase resistance protein thermal stability [50], [51]. Besides, the *k*_cat_/*K*_m_ values of these two mutants were approximately five- and two-fold greater than that of the wild-type, respectively (Table 3). A similar observation was reported that the inter-subunit salt bridge of quinone reductase obtained from *Bacillus subtilis* affects not only protein oligomerization but also catalytic activity [51].

In the present study, the weighted electrostatic attraction model could be used to calculate the interaction between two oppositely charged residues to identify the preferred pairings and the possible substitution in a salt-bridge design. The most stable electrostatic pair at each of the three identified positions increased the BglA thermal stability by charge addition, charge reversion, and polarity reversal. The computational prediction scores clearly identified the positively or negatively charged substitutions with increased *T*_m_, which suggested that the model could be useful in the design of thermostable proteins.

Accordingly, various rational techniques have been employed to improve protein thermal stability, including consensus-guided mutagenesis and the comparison of thermophiles and mesophilic homologues [52]–[55]. Most of these methods adopt the evolutionary analysis of multiple sequence alignment to substitute the conserved residue for the non-conserved ones at an aligned position or to substitute a residue from thermophiles for a residue from mesophiles. The bioinformatics method used in this study managed to recognize the potential charge substitutions for the low conservation residues from the surrounding electrostatic environment.

## Conclusion

Our approach recognized effective residues and suitable substitutions for designing salt bridges to improve protein thermal stability. Statistical analysis of 10,556 surface salt bridges on 6,493 X-ray protein structures was performed to reveal the preferred geometric characteristics of specific types of salt bridges. The designed weight matrix was used in a weighted electrostatic attraction model to identify the effective pairings. We successfully designed electrostatic pairs at five positions on a mesophilic β-glucosidase, which was experimentally verified. These individual electrostatic pairs may also provide insights into the thermal adaptation exhibited by salt bridges. Combining three electrostatic pairs generated an increase of 15.7°C in *T*_m_. Thus, the method varied from the existing popular thought and could provide a simple alternative to increase the thermal stability of proteins.

## Supporting Information

Figure S1 **rWCN (black line) and B-factor (dotted line) profiles of 1BGA. A.** The positions for mutations (i.e., E96, Q141, T231, Q277, N437, S440 and Q216) are indicated with hollow circles and diamonds corresponding to their z_rWCN and z_B-factor values, respectively. If two symbols are overlapped, only circle is indicated. The residues in the interface regions are indicated by black (A/D) and gray (A/E) thick lines on the horizontal axis. rWCN and B-factor values are normalized to their respective Z-scores (z_rWCN and z_B-factor values).
(TIF)

Figure S2 **Melting curves of BglA and mutant proteins.** The mutants showed in the legend are listed in the order of their *T*m values from high to low. The melting curves of (A) T231, Q277, (B) E96, N437, S440, (C) Q216, and Q141 mutants as well as the mutant containing the three thermostable pairs (E96R–D28, N437K–D49, and T231D–D388K) were compared with that of the wild type.
(TIF)

Table S1 **Empirical weight index of salt-bridge pairing preferences.** These weight values (*w_ij_*) corresponds to the frequency of the specific types of salt bridges in a total of 10,556 surface salt bridges classified by residue pairs, Cα–Cα distances, and secondary structure locations. The sum of all the values equals to 100.
(XLSX)

Table S2 **Characteristics of 447 residues of BglA for the pre-filtering.** The parameters demanded in the pre-filtering contains the structural factors (RSA, B-factor, rWCN) and the conservation score obtained from obtained from the Consurf server.
(XLSX)

Table S3 **A total of 34 residues fit the pre-filtering criteria in BglA.** The listed 34 residues fit the pre-filtering criteria as follows: polar or charge amino acids (STCNQ/KRDE), RSA>35%, z_bf>0, z_rWCN>0, and sequence conservation <4.
(XLSX)

Table S4 **Identification of potential pairs for each 34 qualified positions.** The positions that possess at least one pairing qualified the angular and sequence separation constrains could be identified.
(XLSX)

Table S5 **Prediction of the charge-charge interactions between each 11 positions and their surrounding residues by scoring each interaction with Eq. (1).** The predicted pair equivalent to the highest-score pair of each position; the suggested amino acids for mutations were the one that fit the predicted pair at each position. If a position’s highest-scored pair is a *i*–*j* <5 pair, then the corresponding mutation is not suggested. An alternative charged substitution is adopted if that substitution for the alternative pair (*i*–*j* ≥5) formation was scored higher than the same substitution for the *i*–*j* <5 pairs. The highest-scored pair is inferred if two or more of such alternative pairs are found.
(XLSX)

Text S1 **Data set for determining geometric preferences of salt-bridges.** The data set contains 32,096 salt bridges isolated from 6,493 protein structures. Four types of salt-bridge pairs were considered: K–D; K–E; R–D; and R–E.
(TXT)
