# Supplementary figures and images for: Protein Thermal Stability Enhancement by Designing Salt Bridges: A Combined Computational and Experimental Study
Source: PLoS One. 2014 Nov 13;9(11):e112751. doi: 10.1371/journal.pone.0112751 (PMC4231051; doi:10.1371/journal.pone.0112751)

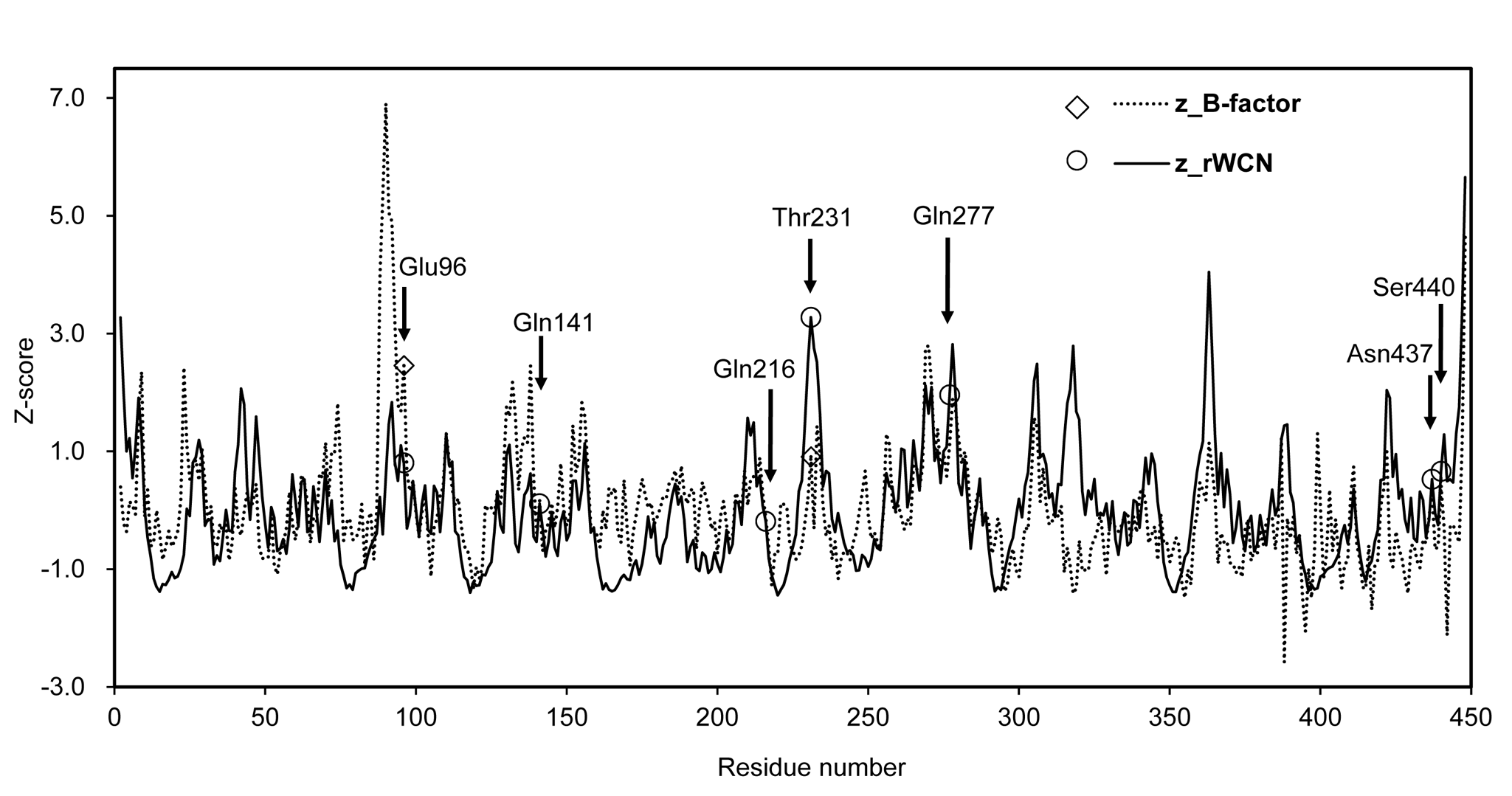

Supplement: Figure S1 — rWCN (black line) and B-factor (dotted line) profiles of 1BGA. A. The positions for mutations (i.e., E96, Q141, T231, Q277, N437, S440 and Q216) are indicated with hollow circles and diamonds corresponding to their z_rWCN and z_B-factor values, respectively. If two symbols are overlapped, only circle is indicated. The residues in the interface regions are indicated by black (A/D) and gray (A/E) thick lines on the horizontal axis. rWCN and B-factor values are normalized to their respective Z-scores (z_rWCN and z_B-factor values). (TIF) [file pone.0112751.s001.tif]

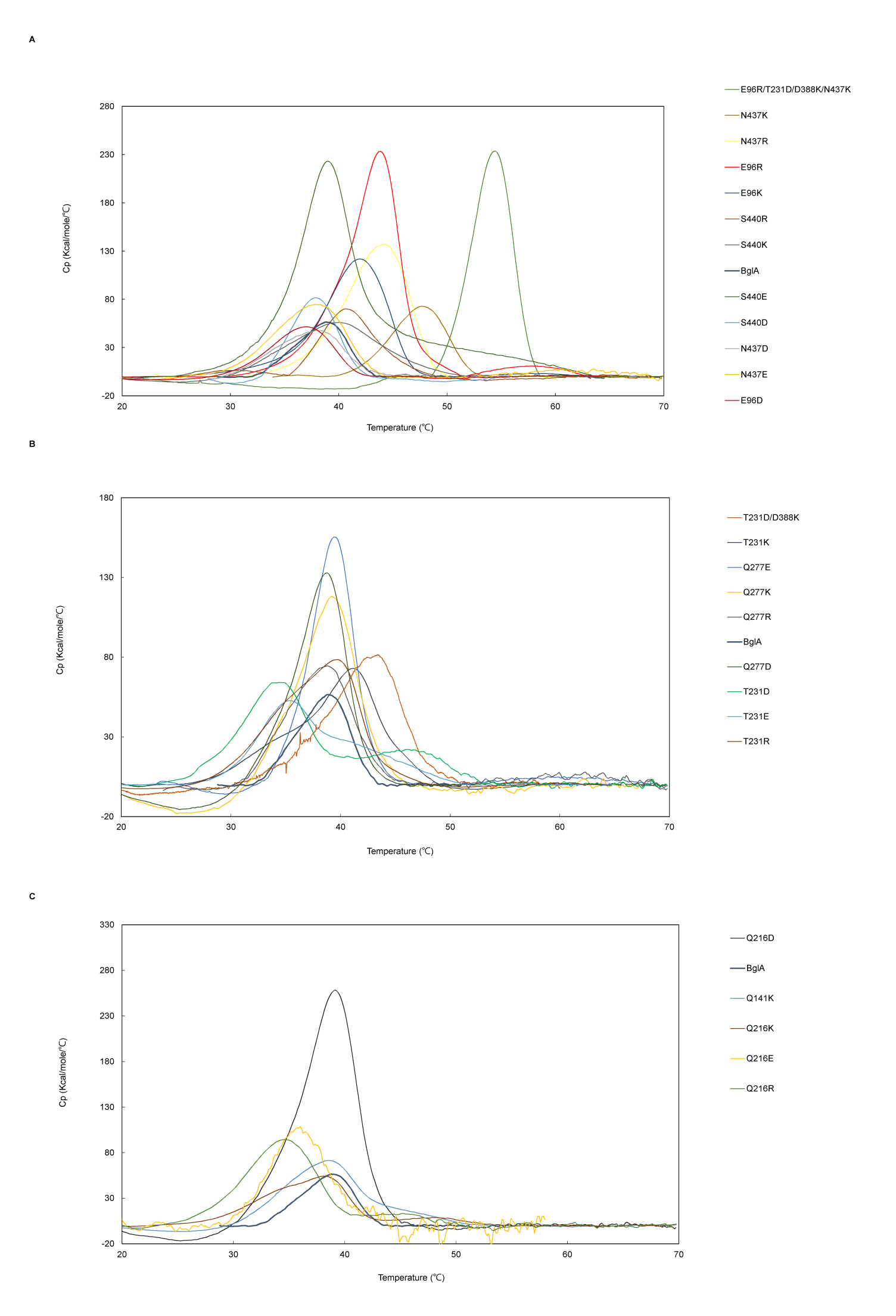

Supplement: Figure S2 — Melting curves of BglA and mutant proteins. The mutants showed in the legend are listed in the order of their Tm values from high to low. The melting curves of (A) T231, Q277, (B) E96, N437, S440, (C) Q216, and Q141 mutants as well as the mutant containing the three thermostable pairs (E96R–D28, N437K–D49, and T231D–D388K) were compared with that of the wild type. (TIF) [file pone.0112751.s002.tif]
